# Supplementary material for: Circulating microRNA Profiles Identify a Patient Subgroup with High Inflammation and Severe Symptoms in Schizophrenia Experiencing Acute Psychosis
Source: Int J Mol Sci. 2024 Apr 12;25(8):4291. doi: 10.3390/ijms25084291 (PMC11050142; doi:10.3390/ijms25084291)
Supplement: Supplementary file 1 [file ijms-25-04291-s001.zip › Supplementary Figures.pdf]

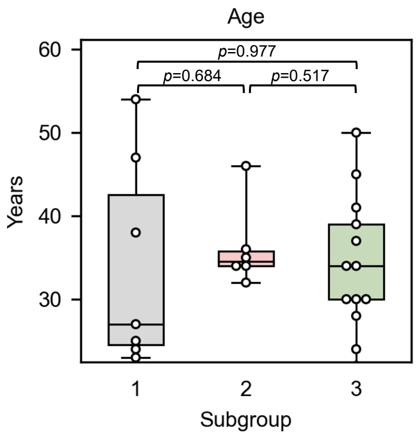

Figure S1. Patients' age was not different among the miRNA-based subgroups. Box-and-swarm plots show patients' age in each subgroup. Welch's *t*-test was applied to compare patients' age among the subgroups (nominal *p*>0.05).

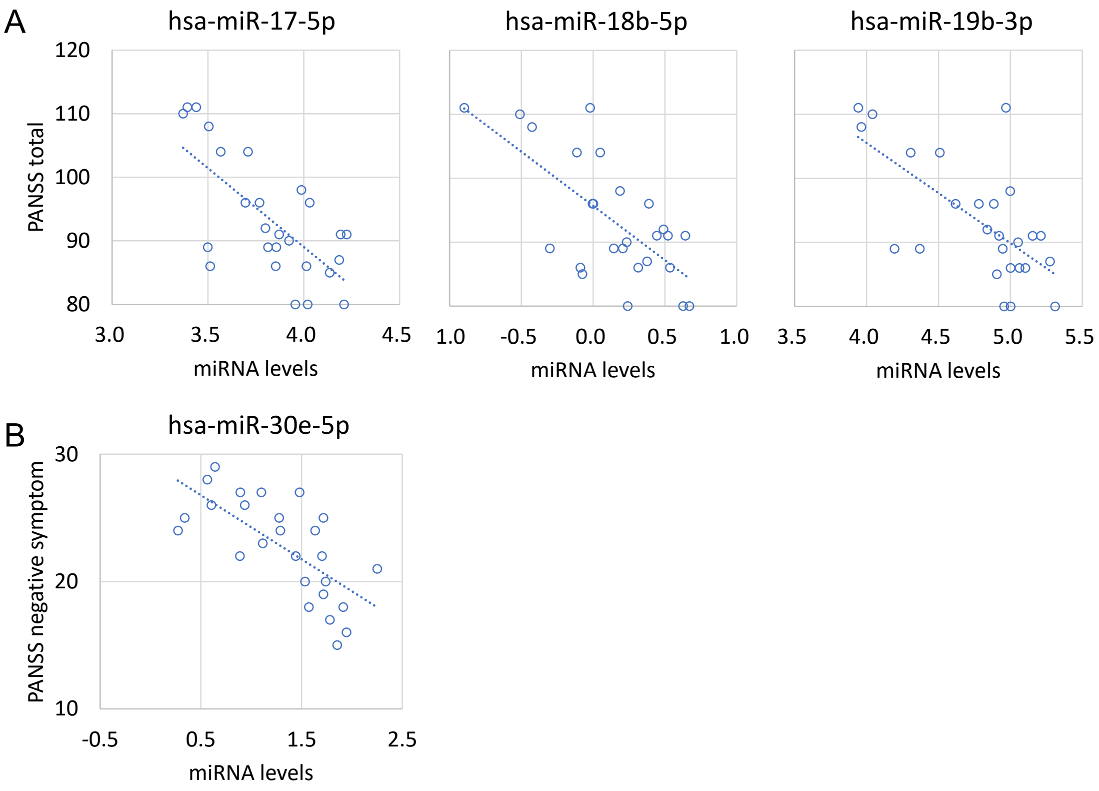

Figure S2. Individual miRNAs were inversely correlated with PANSS scores in the 26 schizophrenia patients. Scatter plots visualize the relationshiops between individual miRNAs and PANSS scores with significant Pearson's correlation coefficients (Benjamini–Hochberg corrected *p*<0.05). (A) correlations between hsa-miR-17-5p, hsa-miR-18b-5p, and hsa-miR-19b-3p with PANSS total scores. (B) a correlation between hsa-miR-30e-5p with PANSS negative symptom subscales.

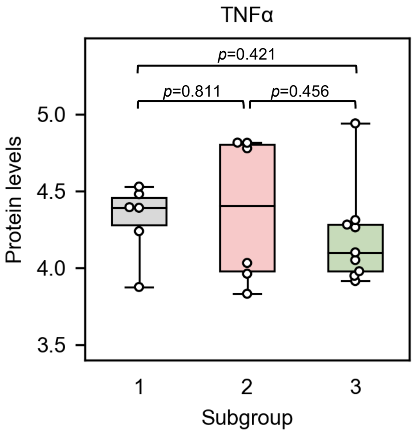

Figure S3. Protein levels of TNFα in plasma were not different among the miRNA-based subgroups. Box-and-swarm plots show expression levels of a TNFα protein in each subgroup. Welch's *t*-test was applied to compare TNFα protein levels among the subgroups (nominal p>0.05).
